# Are experiences of family and of organized violence predictors of aggression and violent behavior? A study with unaccompanied refugee minors

**DOI:** 10.3402/ejpt.v7.27856

**Published:** 2016-02-12

**Authors:** Veronika Mueller-Bamouh, Martina Ruf-Leuschner, Katalin Dohrmann, Maggie Schauer, Thomas Elbert

**Affiliations:** 1 Department of Psychology, University of Konstanz, Konstanz, Germany; 2 vivo international e.V. (www.vivo.org), Germany

**Keywords:** Violence, conflict, war, aggression, refugee minors, adolescents, PTSD, trauma exposure, traumatic events

## Abstract

**Background:**

There is strong support for familial abuse as a risk factor for later delinquency and violent offending, whereas empirical evidence about the contribution of experienced organized violence to the cycle of violence is less clear. Nevertheless not all abused children do become violent offenders. This raises the question of which factors influence these children's risk of future aggressive behavior. Recent evidence suggests that the trait of appetitive aggression plays an important role in the prediction of aggressive behavior.

**Objective:**

The focus of the study is to investigate whether exposures to 1) organized; and 2) family violence equally contribute to aggressive behavior and how this is related to a trait of appetitive aggression. Furthermore it is of interest to uncover how the severity of posttraumatic stress disorder (PTSD) symptoms modulates associations between violent experiences and aggression.

**Method:**

To answer these questions, we investigated unaccompanied refugee minors who had been exposed to varying levels of both violence types. Using structured interviews, experiences of organized and familial violence, self-committed aggressive acts, the trait of appetitive aggression, and PTSD symptoms were assessed in 49 volunteers.

**Results:**

A sequential regression analysis revealed that the trait of appetitive aggression and experienced family violence were independent and significant predictors of self-committed aggressive acts, altogether accounting for 70% of the variance. Exposure to organized violence, however, was not significantly associated with aggressive acts or appetitive aggression. PTSD symptom severity was not correlated with measures of aggression but with the exposure to familial and organized violence.

**Conclusions:**

Results suggest that in addition to the impact of family violence, an elevated trait of appetitive aggression plays a crucial role in aggressive behavior and should be considered in psychotherapeutic treatment.

Studies in various settings illustrated familial childhood maltreatment and corporal punishment to be associated with later aggressive behavior, delinquency, and violent offending (e.g., Gershoff, 2002; Hecker, Hermenau, Isele, & Elbert, 2013; Mersky, Topitzes, & Reynolds, 2011; Widom & Maxfield, 2001). However, these problems in adulthood vary in intensity and a large portion of abused children do not become violent offenders (e.g., Elbert, Rockstroh, Kolassa, Schauer, & Neuner, 2006; Widom & Maxfield, 2001). This raises the question of which factors influence these children's risk of future violent behavior.

Haj-Yahia and Abdo-Kaloti (2007), who investigated adolescents living in the Palestinian Authority, showed that multiple self-experienced and witnessed physical, psychological, and verbal abuse within the family in childhood and adolescence predict delinquent and aggressive behavior. Moreover, high levels of political stressors, poor housing conditions, and psychological adjustment problems of the parents increased the negative consequences of exposure to family violence regarding mental health, aggression, and delinquency. Political stressors and violence in crisis regions might enhance violence within families. Stress, anger, and irritability resulting from posttraumatic stress disorder (PTSD) are predicted to lead to poor child rearing, but problem behavior in children as a consequence of PTSD might also play a role (Catani, 2010; Ruf-Leuschner, Roth, & Schauer, 2014). Stimmel and colleagues for example reported PTSD symptom severity to be positively related to reactive aggression (Stimmel, Cruise, Ford, & Weiss, 2014).

In the following study, we asked whether the exposure to organized and familial violence might be amplification factors of aggressive behavior and whether PTSD is influencing the model.

Aggressive behavior can be categorized according to its function and motivation: *Reactive aggression* is a defensive response to danger and threat, and is characterized by high arousal and impulsive responding. If successful, the negative affect caused by the perceived threat will wane and the behavior is thus rewarding. *Proactive aggression*, in contrast, is initiated to obtain specific material gains or a higher social status which then act as rewards (Vitaro, Brendgen, & Barker, 2006; Vitiello & Stoff, 1997). However, aggressive behavior can also be intrinsically rewarding. Independent of its instrumental aspect, it can be experienced as exciting and fascinating (Crombach & Elbert, 2014; Elbert, Weierstall, & Schauer, 2010; Hecker, Hermenau, Maedl, Elbert, & Schauer, 2012). This intrinsically motivated appetite for aggression can spur individuals to seek or create conditions in which they can act out violently. Computer games, hunting, or sports are socially acceptable activities that provide an outlet for this form of aggression. Active aggression against humans requires overcoming socially conditioned inhibitions against violence. This violence-related enjoyment has been termed *appetitive aggression* (Elbert et al., 2010).

Aggressive conduct is influenced by early socializing experiences, situational factors, and biological components (Anderson & Carnagery, 2004). Childhood experiences are particularly important, as during childhood moral development occurs and brain plasticity is at its greatest (Elbert et al., 2006). In consequence of societal laws and customs, mainly instilled by the parents, children build up control and inhibition of extreme forms of violence (Elbert et al., 2010). Most children begin early in learning alternatives to physical aggression (Tremblay, 2010). In societies where violence has become a part of everyday life, this control may not be taught and a high appetite for aggression can even become adaptive, as it may help people to survive in a harsh and unpredictable social environment (Crombach & Elbert, 2014). For example aggressors in a violent environment are often conferred higher social status (Crombach, Weierstall, Hecker, Schalinski, & Elbert, 2013). Moreover, a disposition towards higher appetitive aggression seems to offer a protective shield against the development of PTSD, as shown for various conflict scenarios (Crombach & Elbert, 2014; Weierstall, Huth, Knecht, Nandi, & Elbert, 2012). Children learn through observation of violence within the family, the community, and potentially society at large, that aggressive behavior is an appropriate way to counter danger and to solve conflicts (Bandura, 1973; Widom, 1989). Self-control theory argues that positive parenting fosters the development of self-control, an ability that allows the inhibition of violent behavior. Shame (violation of internalized cultural norms) and guilt (violations of one's own values) arise when norms are violated and prevent morally unjustified behavior (Gottfredson & Hirschi, 1990).

Although there is strong support for the cycle of violence hypothesis for familial abuse, the effects of the exposure to organized and state-sponsored violence are less clear. For children and adolescents growing up in unstable regions, self-experienced and witnessed violent experiences outside their families are commonplace. Qouta and his colleagues found in a study with Palestinian school children in the Gaza Strip, that witnessing military violence was a significant predictor for reactive and proactive aggression as well as enjoyment of aggression. Being a victim of military violence was associated with reactive aggression (Qouta, Punamäki, Miller, & El-Sarraj, 2008).

An investigation of elementary school students in Croatia, several years after the war, reported that exposure to war events increased aggressive behavior, which included reactive and proactive aggression (Kerestes, 2006). Another study of Lebanese children however, failed to observe an effect of war events on aggressive behavior (Macksoud & Aber, 1996).

Empirical evidence about the contribution of experienced organized violence is rare and conflicting. With the present study we attempted to determine the contributions of familial and organized violence to the development of aggressive behavior.

Unaccompanied refugee minors (URM), driven out of their homeland due to persecution, violence, and lack of basic resources for survival, experience both sources of violence. They are forced to seek a new host country alone, without parents or caregivers. Studies point out that URM have endured more traumatic events, especially war events, loss of important persons, and physical and sexual maltreatment, than minors fleeing with their parents (Bean, Derluyn, Eurelings-Bontekoe, Broekaert, & Spinhoven, 2007; Huemer et al., 2009). URM suffer more frequently from PTSD, anxiety, and depression symptoms than accompanied minor refugees (Huemer et al., 2009). Findings concerning externalizing problems in URM are inconclusive (Bean et al., 2007; Bronstein, Montgomery, & Ott, 2013; Oppedal & Idsoe, 2012; Thommessen, Laghi, Cerrone, Baiocco, & Todd, 2013).

For the present study, we predicted that both types of violent experiences—familial and organized—would be significant predictors of aggressive behavior, which we assessed by self-report. Furthermore we examined associations of family violence, organized violence, and PTSD symptom severity, with self-committed violent acts and the trait for appetitive aggression. Because PTSD often results in hypervigilance, irritability, and anger, PTSD symptom severity might modulate the relationship between experiences of violence and self-reported aggression. Additionally experiences of organized violence might be associated with familial violence. We predicted that the positive relationship between self-committed violent acts and appetitive aggression, reported previously, will be replicated.

## Method

### Procedure

To recruit participants, letters stating the aims and the procedure of the study were sent to different child and youth care institutions, refugee centers, non-governmental organizations and legal guardians of youth welfare services. The letter clarified that the participation would be voluntary at every stage of the investigation, costs for travelling and translation would be covered, and a total of 15 Euros would be offered as compensation for the total time needed (travel and interview). Social workers in charge were requested to inform adolescents up to the age of 21, who had entered Germany as minors without parents or other caregivers, about the project. All individuals who met these criteria could participate in the study. The institutions were contacted by phone 2–3 weeks after the written information had been sent. With the consent of the legal guardians, interested URM were invited to attend a structured interview. Eleven clinical psychologists working for the Center of Excellence for Psychotraumatology conducted the interviews. First, the purpose and the procedure of the study were explained in detail to the participants. The interviewer confirmed that the data gathered would be treated confidentially. Furthermore, the participants were assured that they could contact the team if they had any additional questions or further problems. All participants gave their written informed consent. Fifty-four percent of the participants travelled to the University of Konstanz for the assessment; for the other cases, the interviews took place at cooperating institutions. Fifty-six percent of the interviews were conducted with the help of trained translators. The remaining interviews were carried out in English or German. The Ethical Review Board of the University of Konstanz approved the study.

An interview was started with 54 male subjects. One interview was terminated by the investigator when it became obvious that the participant was suffering from acute psychosis. Four participants did not complete the interview either due to a lack of time or because they declined to answer all questions.

### Measures

All instruments were applied in interview form. First *sociodemographic information* including age, living situation, country of origin, date of leaving the home country, date of arrival in Germany, residence status, family situation, and education were assessed. In addition to aggression, violent experiences, and PTSD symptoms, other comorbid problems including depression, suicidality, psychosomatic problems, strengths and difficulties, parents’ child rearing behavior, and disorders like substance abuse and dependency, conduct disorder, and oppositional defiant disorder were assessed (but are not further reported here).

#### Family violence

Information on violence experienced within the family was obtained using the *Checklist of Family Violence* (Ruf & Elbert, 2012). The instrument includes experiences of physical, emotional, and sexual abuse as well as neglect and witnessed violence between family members. Each of the 36 items measures whether one event type has been experienced or not. Additionally, it assesses injuries or the need for medical help in consequence of violent experiences, and the subjective criteria of a traumatic event with reference to the DSM-IV. The sum score of family violence includes all witnessed and self-experienced events. It does not consider how often one type of violence has been experienced. Possible scores range from 0 to 36.

#### Organized violence

In order to assess organized violence, the short version of the *vivo international* checklist of war, detention, and torture events was used (Schauer, Neuner, & Elbert, 2011). The checklist contains 19 different torture event types and 6 war-related events. Each item asked if a particular event was experienced or not (e.g., “Have you been regularly prevented of sleeping?”, “Have you seen people with mutilations or dead bodies?”). The sum score reports how many different types of organized violence have been experienced. Possible scores range from 0 to 25.

#### Self-committed aggressive acts and appetitive aggression

Violence-related enjoyment and self-committed aggressive acts were assessed with the *Appetitive Aggression Scale for children* (AAS-C; Crombach & Elbert, 2014). This questionnaire is based on the AAS for combatants (Weierstall & Elbert, 2011) and includes 17 items assessing the appetitive perception of aggression. With the aid of a Likert scale from 0 (“I do not agree”) to 4 (“I totally agree”) the participants rate how much they agree with each item (e.g., “Is it more fun to harm others if it is more difficult to defeat them?”). The sum score of the rating represents appetitive aggression and could range from 0 to 68. Cronbach's Alpha coefficient was α=0.87 in the present sample.

To quantify self-committed aggressive acts, a checklist with 18 different items was used. This checklist is the first part of the AAS-C and includes different aggressive actions (e.g., “Have you ever hit back when being attacked?”, “Have you ever destroyed someone else's property to tease him/her?”) as well as aggressive fantasy (e.g., “Have you ever imagined in detail how it would be to torture another person physically?”). The possible sum score of committed aggressive acts ranges from 0 to 18.

#### PTSD symptom severity

To assess PTSD symptoms, the *University of California Los Angeles* (UCLA) *PTSD Index for children and adolescents* (Ruf, Schauer, & Elbert, 2011; Steinberg, Brymer, Decker, & Pynoos, 2004) was used in interview form. In the first part of the interview different traumatic experiences are recorded with the help of a 12-item trauma checklist. For the worst event, the A-criterion for a traumatic event, according to the DSM-IV, is assessed. PTSD symptoms are assessed in the second part. The frequency of the symptoms is rated on a Likert scale from (0) *none of the time* to (4) *most of the time*. An overall PTSD symptom severity score can be calculated by summing up the symptom scores. The possible maximum score is 68. The UCLA PTSD Index shows good psychometric qualities and has been used in different cultural settings (Catani, Jacob, Schauer, Kohila, & Neuner, 2008; Crombach & Elbert, 2014; Ruf, Schauer, & Elbert, 2010). Cronbach's Alpha for the PTSD symptoms scale for this sample was α=0.95.

### Data analysis

Statistical analyses were executed with SPSS 21.0. In order to examine the contributions of experienced family violence, organized violence, and appetitive aggression to self-committed aggressive acts, a multiple sequential regression analysis was conducted. Based on previous reports, variables were ranked and included based on their estimated explanatory importance. In the first step, only appetitive aggression was entered as a predictor. In the next step, experienced family violence was added, and in the last step, organized violence and the standardized product of organized and familial violence were included in the model. All relevant criteria for regression analyses were fulfilled. No problematic outliers were identified. Cook's Distance reached a Maximum of 0.184. The standardized residuals were approximately normally distributed. The variance inflation factor was satisfactory, being close to 1. Homoscedasticity criteria were satisfied. To provide a better understanding of the role of PTSD, associations between the PTSD symptom severity score, violent experiences (familial and organized), and aggression variables were investigated. Correlations between variables were calculated using Pearson's correlation coefficients.

## Results

### Sociodemographic background

An overview of sociodemographic details is shown in Table 1. The remaining 49 male participants were between 13 and 21 years of age with an average age of 17.37 years (*SD=*1.35). On average they had lived 15.57 (*SD=*20.63) months in Germany. The URM participating originated from 17 different countries. Most came from Afghanistan 36.7% (*n=*18), followed by Iraq and Nigeria (each 10.2%, *n=*5), 6.1% (*n=*3) were from Somalia and Gambia, 4.1% (*n=*2) from Iran, Syria, and Sierra Leone, and 2% (*n=*1) from Angola, Pakistan, Azerbaijan, Tunisia, Turkey, Sri Lanka, Palestine, Algeria, and Morocco. Of the total sample, 36.8% (*n=*18) reported that one of their parents was missing or dead, and 40.8% (*n=*20) had lost both of their parents.

**Table 1:** Descriptive statistics of the sample

		(n) %	M (SD); Range
Age in years		–	17.37 (1.35); 13–21
Country of origin	Afghanistan	(18) 36.7	–
	Iraq	(5) 10.2	–
	Nigeria	(5) 10.2	–
	Somalia	(3) 6.1	–
	Gambia	(3) 6.1	–
	Iran	(2) 4.1	–
	Syria	(2) 4.1	–
	Sierra Leone	(2) 4.1	–
	Other	(9) 18.7	–
Months lived in Germany		–	15.57 (20.63); 1–132
Years in school		–	5.46 (3.58); 0–14.7
Parents alive?	Both parents alive	(11) 22.4	–
	One parent dead/missing	(18) 36.8	–
	Both parents dead/missing	(20) 40.8	–
Living situation	Youth care centers	(21) 42.9	–
	Reception centers for URM	(13) 26.5	–
	Centers for asylum seekers	(8) 16.3	–
	Foster families	(5) 10.2	–
	Lived alone	(2) 4.1	–
Residence status	Applied for asylum	(30) 61.2	–
	Not yet applied for asylum	(13) 26.6	–
	Suspension of deportation	(3) 6.1	–
	Residence permit	(3) 6.1	–
Family violence		–	9.04 (7.14); 0–27
Organized violence		–	5.93 (5.58); 0–23
Self-committed violent acts		–	4.39 (4.23); 0–16
Appetitive aggression		–	10.1 (11.02); 0–38
PTSD symptom severity		–	19.31 (17.18); 0–57

*M*=mean, *SD*=standard deviation.

The URM participating lived in 18 different institutions: 42.9% (*n=*21) reported living in youth-care centers, 26.5% (*n=*13) lived in reception centers for URM, 16.3% (*n=*8) stayed in centers for asylum seekers, 10.2% (*n=*5) resided in foster families, and two URM lived by themselves. On average, participants had attended school for 5.46 years (*SD=*3.58) whereas 15.6% (*n=*7) had never been to school. Regarding the residency status, 61.2% (*n=*30) had applied for asylum, 26.6% (*n=*13) had not yet sought asylum, and 6.1% (*n=*3) each had a suspension of deportation or a residence permit.

### Exposure to violence and self-committed aggressive acts

The majority of URM (91.8%; *n=*45) reported at least two events of family violence and 40.8% (*n=*20) suffered from injuries due to family violence. On average nine different events of family violence were stated (*M=*9.04, *SD=*7.14).

In our sample, 42.9% (*n=*21) experienced at least one torture event during detention or abduction through police, soldiers, or rebels, whereas 81.6% (*n=*40) reported at least one war event, like witnessing death or injuries of strangers or family members, or bombardments. Six different event types of organized violence were reported on average (*M=*5.93, *SD=*5.58). Taken together, only 16.3% (*n=*8) did not report any experience of organized violence. On average, participants committed four different violent acts (*M=*4.39, *SD=*4.23), like fighting back when being attacked (67.3%, *n=*33), attacking somebody (42.9%, *n=*21), or threatening (36.7%, *n=*18) somebody else. An overview of different self-committed aggressive acts is presented in Table 2.

**Table 2:** Percentages of different committed aggressive acts

Self-committed acts	(n) %
Hit back when being attacked	(33) 67.3
Defend oneself in a fight	(32) 65.3
Physically attacked another person	(21) 42.9
Threatened or bullied someone	(18) 36.7
Imagined in how it would be to torture another person	(15) 30.6
Made another person bleed	(14) 28.6
Talked with others about experiences of harming others	(13) 26.5
Destroyed something of another person to tease him/her	(11) 22.4
Injured another person with a weapon	(10) 20.4
Taken things from others against their will	(10) 20.4
Made another person scream out of pain	(9) 18.4
Instructed others to harm another person	(9) 18.4
Had the wish to once be involved actively in a fight	(9) 18.4
Chased another person that you wanted to harm	(8) 16.3
Injured another person dangerously or killed another person	(6) 12.2
Used physical force to get others to do what you want	(5) 10.2
Harmed another person that could not defend him/herself	(5) 10.2
Sexually assaulted another person	(1) 2.0

### Family and organized violence as predictors for self-committed aggressive acts

In a multiple sequential regression analysis, self-committed aggressive acts are regressed on appetitive aggression, experiences of family violence, and organized violence (Table 3). The first model with appetitive aggression as the only predictor explained 65% of variability in different self-committed aggressive acts (*R*^2^= 0.66, *F*_(1, 47)_=91, *p*<0.001). After adding family violence as second predictor, the model accounted for 70% of variation in reported aggressive acts. The change of *R* ^2^ was significant (Δ*R* ^2^ *=*0.05; *F*_(1, 46)_=7.87, *p*<0.01). Organized violence as a new predictor in the third step did not improve the model significantly (Δ*R* ^2^=0.02; *F*_(2,44)_=1.5, *p=*0.23) and thus could not significantly contribute to the prediction of self-committed aggressive acts. Because the interaction term was also not significant, there was no moderation effect of organized violence on the effect between family violence and self-committed aggressive acts.

**Table 3:** Sequential regression analysis predicting self-committed aggressive acts

	Self-committed aggressive acts				
			
	*B*	SE	ß	*T*	*R*^2^
Step 1					
Appetitive aggression	0.31	0.03	0.81	9.54***	0.66
Step 2					
Appetitive aggression	0.28	0.03	0.72	8.25***	0.71
Family violence	0.14	0.05	0.24	2.81**	
Step 3					
Appetitive aggression	0.28	0.03	0.73	8.47***	0.73
Family violence	0.14	0.05	0.24	2.82**	
Organized violence	−0.69	0.06	−0.09	−1.13	
Family violence×organized violence	−0.64	0.41	−0.13	−1.54	

*B*=unstandardized regression weight; *SE*=standard error; ß=standardized regression weight; *T*=*t*-test statistics,

*** *p*<0.001;

** *p*<0.01.

### Correlations between violent events, PTSD, and measures of aggression

**Fig. 1:**
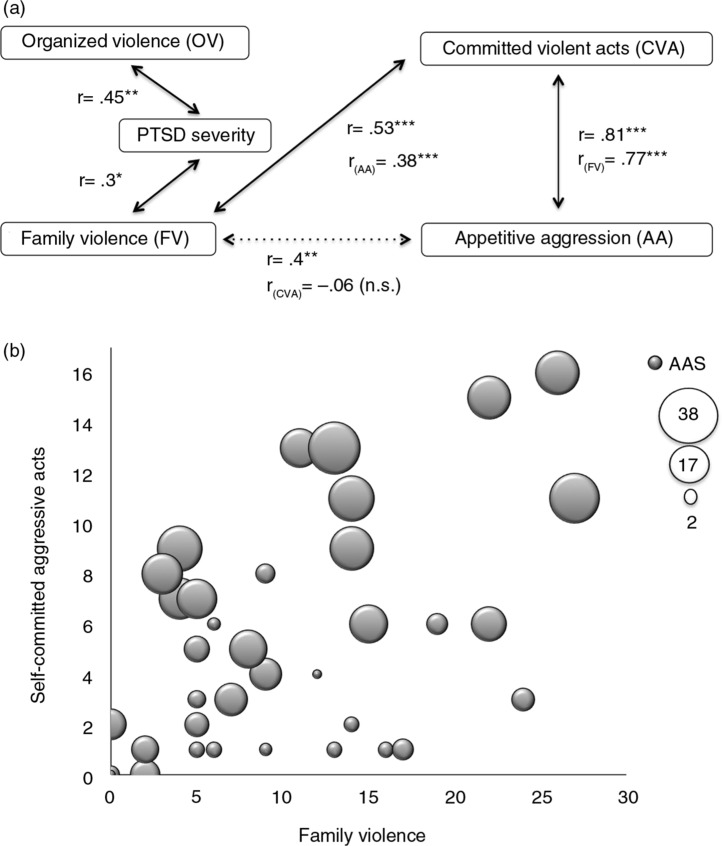
(a) Correlation coefficients between all variables were calculated. Only significant correlations are pictured (n.s.=not significant; *r*=Pearson's correlation coefficient; *r*_(OV)_=*r* (controlled for organized violence)=partial correlation coefficient; *p<0.05; **p<0.001; ***p<0.001). (b) A 3D-scattergram, showing that the number of committed violent acts increases with the amount of family violence experienced. It further demonstrates that appetitive aggression also increases. The magnitude of the appetitive aggression score is indicated by the size of the bubbles.

Pearson's correlation coefficient was calculated to detect relevant relationships between variables. In Fig. 1a significant correlations are reported.

Experienced family violence was positively associated with self-committed aggressive acts and with appetitive aggression, whereas self-committed aggressive acts and appetitive aggression were also highly correlated. Experiences of organized violence however were not associated with committed violence (*r=*−0.06, *p=*0.71) or appetitive aggression (*r=*0.01, *p=*0.92). In Fig. 1b the correlation between experienced family violence and self-committed aggressive acts are plotted. The size of the bubbles additionally illustrates the magnitude of the appetitive aggression score.

There was no significant relationship between PTSD symptom severity; and self-committed violent acts (*r=*0.14, *p=*0.34), nor was there any correlation between appetitive aggression and PTSD symptom severity (*r=*0.06, *p=*0.67). PTSD severity was significantly higher when participants reported more organized violence or more family violence. Experiences of organized violence were not significant related to experiences of family violence (*r=*−0.01, *p=*0.96).

## Discussion

In this study the links between violent experiences—organized and family violence—with aggressive behavior and violence-related enjoyment were examined in URM. As hypothesized, the number of different violent experiences within family was positively related to the number of reported aggressive acts. This result is in line with social learning and self-control theory, which argue that bad child rearing inhibits the development of self -control, makes violent behavior acceptable, and prevents the development of internal norms (Bandura, 1973; Gottfredson & Hirschi, 1990). Family violence as a risk factor for later aggressive behavior and delinquency is well documented in the literature (e.g., Mersky et al., 2011). However social learning theory and self-control theory are not able to account for the fact that some abused children become violent offenders while others do not offend, and some non-abused children do grow up to engage in violent behavior.

Results of our study show that, apart from the correlation between family violence and self-committed violence, young people with high appetitive aggression scores are at a higher risk of engaging in aggressive acts. The strong relationship between appetitive aggression and self-committed violent behavior is consistent with results in combatants (Hecker et al., 2012), former child soldiers (Crombach, et al., 2013), and street children (Crombach & Elbert, 2014).

The regression analysis revealed that the trait of appetitive aggression was the strongest predictor in the model followed by family violence, which also significantly contributed to the prediction of committed aggressive acts. If children learn through bad parental child rearing, that violence is an acceptable way of solving conflicts and handling negative emotions, then they are more likely to engage in violent behavior. Nevertheless high appetitive aggression is associated with more violent behavior independently of experiences at home. Children and adolescents who experience aggression as highly appetitive and report violence within their families show the highest rates of aggressive behavior.

Aggressive behavior, which might be learned as a strategy to solve problems due to models in the family, can provide feelings of power and control which can intensify enjoyment of violence (Crombach et al., 2013; Hecker et al., 2012). At the same time an elevated trait of appetitive aggression might lead to more aggressive behavior, which possibly leads to more violence within the family. Studies suggest that individuals vary in their dispositions for appetitive aggression and differ therefore in their probability of acting out violently. It can be assumed that violence-related enjoyment and aggressive behavior mutually reinforce each other (Crombach et al., 2013; Hecker et al., 2012). This could be especially true for contexts in which violent behavior is accepted.

We analyzed the differential contribution of exposure to war and other organized violence towards aggressive behavior and violence-related enjoyment. Against our hypotheses, experiences of organized violence were not found to be significantly associated with self-committed violent acts or appetitive aggression. The latter result is in line with the study of Hecker et al. (2012), which also found no significant association between appetitive aggression and witnessed or self-experienced violence. The omnipresence of violence in conflict regions does not appear to facilitate aggressive behavior in our study. The result suggests that the family context is the most meaningful factor for the development of norms and internal values, whereas violent state-sponsored experiences outside the family have no significant influence. Results of the present study are contrary to the results of Qouta et al. (2008), which showed witnessed military violence to be associated with aggressive behavior and enjoyment of aggression. The study assessed children in acute danger whereas Kerestes (2006), studying children in Croatia, found only a very small effect of exposure to war events on aggressiveness several years after the war. Macksoud and Aber (1996) did not find an effect of war and military violence in Lebanese children. One point that should be noted is that our participants mostly emigrated because of organized violence. It might be that children and adolescents who have adapted well to the violent environment and who would show higher scores in aggressive behavior and appetitive aggression are less likely to have left their countries.

Contrary to the suggestion that stress in parents and children due to political and military violence might enhance violence in families (Catani, 2010; Haj-Yahia & Abdo-Kaloti, 2007), exposure to organized violence was neither associated with greater levels of family violence nor did it moderate the effect between family violence and reported aggressive acts. However it is possible that some of the reported organized violent events were experienced after separation from the parents.

Concordant with prior research (e.g., Neuner et al., 2004) the building block effect could be replicated in our study. Both organized and familial violence were significantly associated with PTSD symptom severity. PTSD severity however was not associated with self-committed aggressive acts. Because of our very selective sample it can be assumed that adolescents who seek shelter in a new country perceived the situation in their home countries as threatening and incriminating and could not adapt well to violent environments.

### Limitations

It should be noted, that the sample size limits statistical power to the extent that only larger effects can be detected. The data are correlational in nature and cannot prove causal relationships. Despite confidentiality being assured to all URM and most participants being very open-minded, a bias due to social desirability or to fears of potential negative consequences for the asylum procedure cannot be ruled out. Worries about family due to loss of contact with parents may have had an influence on reports of family violence. An impact of the diverse cultural background of the sample on answering questions cannot be ruled out. For further studies it would be interesting to consider reactively and proactively motivated aggressive behavior, and to differentiate between witnessed and self-experienced violent events. It should be considered that a sample of young unaccompanied refugees is selective, and observed associations deserve replication in other different samples.

## Conclusions

The present study contributes to a better understanding of violent and aggressive behavior. Results suggest another important, and to date unexplored factor, in the cycle of violence: Appetitive aggression turned out to be the most important predictor for self-committed aggressive acts. It is crucial to be considered in therapy. Experiences of organized violence were not associated with aggressive behavior however, both organized violence and family violence contributed to PTSD symptom severity. Therapy with young refugees needs to consider experiences within the family as a contributing factor to clinical problems, and not just traumatic experiences concerning war and state sponsored violence.

The development of the trait of appetitive aggression and its implication for future violent behavior in various contexts should additionally be addressed in longitudinal studies.
